# Association between PM_2.5_ and risk of hospitalization for myocardial infarction: a systematic review and a meta-analysis

**DOI:** 10.1186/s12889-020-8262-3

**Published:** 2020-03-12

**Authors:** Zeynab Farhadi, Hasan Abulghasem Gorgi, Hosein Shabaninejad, Mouloud Aghajani Delavar, Sogand Torani

**Affiliations:** 1 Department of Health Services Management, School of Health Management and Information Sciences, Iran University of Medical Sciences, Tehran, Iran; 2 Department of Health Economics, School of Health Management and Information Sciences, Iran University of Medical Sciences, Tehran, Iran; 3 Infertility and Reproductive Health Research Center, Research Institute for Health, Babol University of Medical Sciences, Babol, Iran

**Keywords:** Fine particulate matter, PM_2.5_, Air pollution, Myocardial infarction, Exposures

## Abstract

**Background:**

It is generally assumed that there have been mixed results in the literature regarding the association between ambient particulate matter (PM) and myocardial infarction (MI). The aim of this meta-analysis was to explore the rate of short-term exposure PM with aerodynamic diameters ≤2.5 μm (PM_2.5_) and examine its potential effect(s) on the risk of MI.

**Methods:**

A systematic search was conducted on databases like PubMed, Scopus, Web of Science, and Embase with components: “air pollution” and “myocardial infarction”. The summary relative risk (RR) and 95% confidence intervals (95%CI) were also calculated to assess the association between the PM_2.5_ and MI.

**Results:**

Twenty-six published studies were ultimately identified as eligible candidates for the meta-analysis of MI until Jun 1, 2018. The results illustrated that a 10-μg/m 3 increase in PM_2.5_ was associated with the risk of MI (RR = 1.02; 95% CI 1.01–1.03; *P* ≤ 0.0001). The heterogeneity of the studies was assessed through a random-effects model with *p* < 0.0001 and the I^2^ was 69.52%, indicating a moderate degree of heterogeneity. We also conducted subgroup analyses including study quality, study design, and study period. Accordingly, it was found that subgroups time series study design and high study period could substantially decrease heterogeneity (I^2^ = 41.61, 41.78).

**Conclusions:**

This meta-analysis indicated that exposure – response between PM_2.5_ and MI. It is vital decision makers implement effective strategies to help improve air pollution, especially in developing countries or prevent exposure to PM_2.5_ to protect human health.

## Background

Air pollution (atmospheric pollution) is the release of harmful particles matter into air by one or more harmful gases. It is generally assumed that any exposure to outdoor particulate matter air pollution can pose a big challenge to both public health agencies and physicians in the world, especially in the developing countries [1]. It is also believed that outdoor air pollution is a threat factor contributing to universal mortality and disability-adjusted life-years (DALYs) which rank the fifth and sixth in the world, respectively [2, 3]. Based on the criteria released by the National Ambient Air Quality Standard (NAAQS), there are six major pollutants of ozone (O_3_), carbon monoxide (CO), lead (PB), sulfur dioxide (SO_2_), nitrogen dioxide (NO_2_), particulate matter < 10 μm (PM_10_), and particulate matter < 2.5 μm (PM_2.5_). Recent findings suggest that any exposure to PM_2.5_ can endanger lungs and blood stream more than other pollutants and can lead to adverse cardiovascular, respiratory, and neurological disorders (Stroke, Alzheimer and Parkinson) as well as premature birth [4–8]. In most countries, it is thought that the level of PM_2.5_ particles is higher than the defined standards, even higher than those set by the World Health Organization (WHO) (WHO) [9, 10]. It is assumed that automobiles and combustion activities are the main sources for the production of PM_2.5_ [11]. Tehran Province in Iran, has been struggling with the highest air pollution in the last few decades, due to fast-growing industrial activities as well as the large number of automobiles on the road [12]. Cohen et al. (2017), in their study, indicated that 103.1 million years of life lost (YLL) and 4.2 million mortality occurred as a consequence of exposure to PM_2.5_.

Myocardial infarction (MI) or cardiac infarction is generally defined as detection of an elevated cardiac troponin (cTn) value which is above the 99th percentile upper reference limit [13]. In recent years, the prevalence of MI has been increased in both developed and developing countries [14, 15]. Research evidences indicate that such risk factors as age, sex, and family history cannot be modulated, but some of the risk factors such as ambient air pollution and unhealthy life style are to a great extent preventable [16–18]. Considering the fact that the age for onset of the first MI has been decreasing and that MI is multifactorial in nature, its fundamental function remains unknown [19].

Therefore, incidence of MI with simultaneous concentration of fine particulate matter has been extensively studied all over the world [20, 21] but only two systematic reviews and meta-analyses about the effect of particulate matter on MI were found Mustafic et al. (2012) revealed that all air pollutants, except for ozone, are significantly correlated with the increased risk of MI. In this meta-analysis, 13 research studies on feature of PM_2.5_ were scrutinized to detect the risk of MI and it was found that the relative risk of overall PM_2.5_ ranked the second after the relative risk of overall carbon monoxide [22]. Moreover, Luo et al. (2015) conducted a meta-analysis based on thirty-one time-series and case crossover studies in order to investigate the effect of particulate matter on the risk of MI. The results demonstrated that the exposure to PM_2.5_ can increase the risk of MI much more than the exposure to PM_10_. The findings also showed that there was a moderate heterogeneity in meta-analysis of the pooled estimates, but the subgroup analyses might not pinpoint the cause of this heterogeneity. Therefore, it is imperative to investigate the source of heterogeneity in a study with more details (Luo et al., 2015) [23]. In this meta-analysis, the rate of PM_2.5_ and the risk of MI are focused. It should be noted that PM_2.5_ was a subgroup in other meta-analysis studies. The time scope in this study is broader as the original studies conducted from January 2000 to June 2018 were attempted to be incorporated. This study has been performed in University of Medical Sciences, Iran in 2018.

## Methods

This protocol was registered in PROSPERO, the International Prospective Register of Systematic Reviews, on 2 January, 2019 (registration number CRD42019118998). The findings of this study were based on the accommodation guidelines: “Preferred reporting items for a protocol for a meta-analysis (PRISMA-P) 2015” [24]. In this study, two reviewers (ZF and MAD) conducted a research on such electronic bibliographic databases including Scopus, Web of Science, PubMed, and EMBASE. They also searched for components such as “air pollution” and “myocardial infarction” and found synonyms using the Medical Subject Headings (MeSH). In addition, the results were combined using the Emtree term and incorporated all other synonyms, except for those found in PubMed. In this way, it was possible to narrow the syntax down to a specific period from Jan. 1, 2000 to Jun. 1, 2018. It is worth mentioning that any study dealing with the short-term relationship between the pollutants and myocardial infarction was thoroughly reviewed. A thorough search on Google Scholar was also performed using dual combinations of the two main components. In an attempt not to miss any study, the grey literature and conference proceedings were explored, and a list of references was ultimately reviewed [25]. There was no language restriction on the search engines. Having completed the search, one of the reviewers (ST) did the duplications using Endnote software version 8 and started to conduct the initial screening through titles and abstracts. Then, the two reviewers reviewed the full-text of the articles carefully for any potentially-relevant studies according to inclusion and exclusion criteria [26]. The inclusion criteria for this meta-analysis allowed for utilization of original studies with time-series or case-crossover designs dealing with any exposure to particulate matter (PM_2.5_), including even a short-term exposure such as the same day or 7 days before the occurrence of MI. The excluded studies had the following traits: 1) not being case-crossover or time-series designs, 2) non-original studies, 3) patients with MI, (4) long-term exposure to particulates matter PM_10_, and 5) no reported relative risk (RR)/odds ratio (OR) and 95% Confidence Interval (CI 95%). Any disagreement in arbitration for the eligibility of the paper was discussed until a consensus was reached by the reviewers.

### Data extraction

The two researchers (H SH and M AD) extracted the data independently using a standardized form which was particularly prepared for studies based on the Cochrane guidelines [27]. The study had to be excluded from meta-analysis in the case of receiving no response from the author. In case of disagreement between the two authors, a third person was called upon as an arbitrator to help reach a consensus. The information in the data extraction form was: name of the author(s), publication year, country, city, study design, study period, lag exposure, case of population, adjustment, effect size, level of exposure to pollution, association between MI and lag exposure (0–6 day), and cumulative lags (0–1, 0–2, 0–3, 0–4, 0–5, 0–6) (Table 1).

**Table 1:** Features of the studies imported to the meta-analysis

No	Author/ publication year	Country	City	Design	Study period (month)	Lag exposure	Case population (n)	Adjustment	Quality score
1	(Peters et al., 2001) [28]	USA	Boston	Case-Crossover	5	Lag0	772	Day of the week, season, and meteorological parameters on the same time scales	High
2	(Peters et al., 2005) [29]	Germany	Augsburg	Case -Crossover	24	Lag0, Lag5, Lag0–4	851	Temperature, humidity, days of the week, pressure	High
3	(Sullivan et al., 2005) [30]	USA	Washington	Case-Crossover	72	Lag0	5793	Relative humidity and temperature	High
4	(Pop et al., 2006) [31]	USA	Utah	Case-Crossover	120	Lag0, Lag3	3910	Temperature	Low
5	(Zanobetti &Schwartz et al. 2006) [32]	USA	Boston	Time Series	48	Lag0, Lag0–1	15,578	Temperature, days of the week	High
6	(Barnett et al., 2006) [33]	Australia	Auckland, Brisbane, Canberra, Christchurch, Melbourne, Perth, Sydney	Case-Crossover	36	Lag0–1	56,036	Day of week, pressure, holidays, temperature, humidity and others	High
7	(Ueda et al., 2009) [34]	Japanese	Fukuoka, Kawasaki, Kobe, Nagoya, Osaka, Sapporo, Sakai, Sendai and Tokyo	Time Series	24	Lag0, Lag1	67,897	Days of the week, seasonality, relative humidity, ambient, and temperature	Low
8	(Stieb et al., 2009) [35]	Canada	Edmonton, Halifax, Montreal, Ottawa, Saint John, Vancouver and Toronto	Time Series	120	Lag0, Lag1, Lag2	63,184	Seasonal cycles, temperature, and humidity	High
9	(Belleudi et al., 2010) [36]	Italy	Rome	Case-Crossover	56	Lag0, Lag6	7520	Influenza, population reduction, epidemics, pressure, and Temperature	Low
10	(Zanobetti &Schwartz 2009) [37]	USA	112 cities (The biggest cities are California, New York City, Los Angeles, Chicago, Illinois and New York)	Time Series	72	Lag0–1	397,894	Long-term trend, seasonality, temperature, days of the week	High
11	(Rich et al., 2010) [38]	USA	New Jersey	Case-Crossover	24	Lag0	5864	Weather and days of the week	High
12	(Berglind et al., 2010)a [39]	Sweden	Boston	Case-Crossover	24	Lag0	772	Relative humidity and temperature	Low
13	(Berglind et al., 2010) b [39]	Sweden	Seattle	Case-Crossover	24	Lag0	5793	Relative humidity and temperature	Low
14	(Berglind et al., 2010) c [39]	Sweden	Augsburg	Case-Crossover	24	Lag0	691	Temperature and relative humidity	Low
15	(Mate et al., 2010) [40]	Spain	Madrid	Time Series	24	Lag6	1096	Days of the week, trend, seasonality, influenza and temperature	High
16	(von Klot et al., 2011) [41]	Germany	Augsburg	Case-Crossover	48	Lag0	960	Days of the week and temperature	High
17	(Chang et al., 2013) [42]	Taiwan	Taipei	Case-Crossover	48	Lag0	14,353	Temperature and relative humidity	High
18	(Rosenthal et al., 2013) [43]	Finland	Helsinki	Case-Crossover	96	Lag0, Lag1, Lag2, Lag3,Lag0–3	629	Temperature and humidity	High
19	(Talbott et al., 2014) [21]	USA	Florida	Case-Crossover	96	Lag0, Lag1, Lag2, Lag0–2	135,421	Maximum apparent temperature and ozone	Low
20	(Gardner et al., 2014) [44]	USA	New York	Case-Crossover	36	Lag0–1,Lag0–2, Lag0–3, Lag0–4	677	Relative humidity and temperature	High
21	(Milojevic et al., 2014) [45]	UK	London	Case-Crossover	72	Lag0–4	452,343	Temperature, days of the week	High
22	(Wichmann et al., 2014) [46]	Sweden	Gothenburg	Case-Crossover	300	Lag0, Lag1, Lag0–1	28,215	Relative humidity, temperature and public holiday	High
23	(Wang et al., 2015) [47]	Canada	Calgary, Edmonton	Case-Crossover	132	Lag(0,1.2.3,4)	22,628	daily average of temperature, dew point temperature and wind speed	Low
24	(Zang et al., 2016) [48]	China	Chaoyang	Case-Crossover	12	Lag(0,1,2,3,4,5)	2749	meteorological conditions and/or other gaseous pollutants	High
25	(Argacha et al., 2016) [49]	Belgian	Belgian	Case-Crossover	48	Lag0	11,428	Day of the week, temperature	High
26	(Baneras et al., 2017) [20]	Spain	Barcelona	Time Series	24	Lag0	4141	Seasonal, meteorological factors, and time-calendar variables	High
27	(Akbarzadeh et al., 2018) [50]	Iran	Tehran	Case-Crossover	24	Lag0–1	208	Temperature and humidity	Low
28	(Yu et al., 2018) [51]	China	Changzhou	Time Series	24	Lag(0,1,2,3,4,5,6), Lag(0–1,0-2,0-3,0-4,0-5,0–6)	5545	Temperature, days of the week, relative humidity, seasonal trends	Low

### Quality score assessment

It is commonly assumed that the quality assessment report for all qualified papers is an indispensable requirement for all case-crossover and time-series studies. Nonetheless, there are currently no valid scales available for assessing the quality of the methodology. To this end, a quality rating scale was adopted and accepted according to the previous meta-analysis (Mustafić et al., 2012). The two reviewers (ST and H AG) managed to evaluate the quality of the study independently based on the following three components [52]. The quality of measurements for ambient concentration PM_2.5_ (0 and 1) was based on the following criteria. Score 0 was recorded in case that the measurements were done under the condition that more than 25% of the data was missing and not taken daily, or showed that there was no description of pollutant measurements. On the other hand, score 1 was recorded in case that measurements were conducted at least once a day, or under the condition that less than 25% of the data was missing. The arrangement of confounders was based on 0 and 1. It is believed that there is a discrepancy between the time-series and the case-crossover studies in their research methods. As a result, the modalities for the arrangement of confounders would be different. Score 1 was recorded in case that the arrangement for covariates was accomplished for multiple main covariates, containing seasonality, temperature, pressure/moisture/day, and long-term processes of week for case-crossover studies which controlled fixed and stilly varying biases using the scheme itself and also for time-series studies. Score 0, however, was recorded for the original papers without modifying the above-mentioned important variables. Finally, if a research study obtained the highest score for all components, it was defined as a high quality one, whereas a study with a minimum score (0 point) for one of the three components was regarded as a low quality one.

### Data synthesis

All studies which examined the relationship between the exposure to PM_2.5_ and MI with relative risk (RR) or odds ratio (OR) and 95% confidence intervals (95%CI) were included. The studies with the statistical estimation risk of MI, relationship with the exposure to PM_2.5_ as OR, and with 95% CI were converted to RR, with 95% CI by using the above formula

$$ RR=\frac{OR}{\left(1-P0\right)+\left(P0\times OR\right)} $$

Since, this meta-analysis aimed to was to explore the rate of short-term exposure on MI. Furthermore, in some original study there was no extended lag patterns of short-term effects of air pollution, thus the exposure lags of 0 or 1 day were selected for calculating the RR. The criteria for heterogeneity were determined through I^2^, and the I^2^ values of more than 50% offered a significant heterogeneity [53]. It should be noted that the fixed-effects models were utilized in case of no heterogeneity. Any potential publication bias was detected through the optical audit of the funnel plots and the Egger regression test. All the analyses were conducted using Comprehensive Meta-analysis Software (Version 2.0, Biostat) and SPSS 24. All the tests were two-tailed tests and *p* < 0.05 was statistically significant.

## Results

**Fig. 1:**
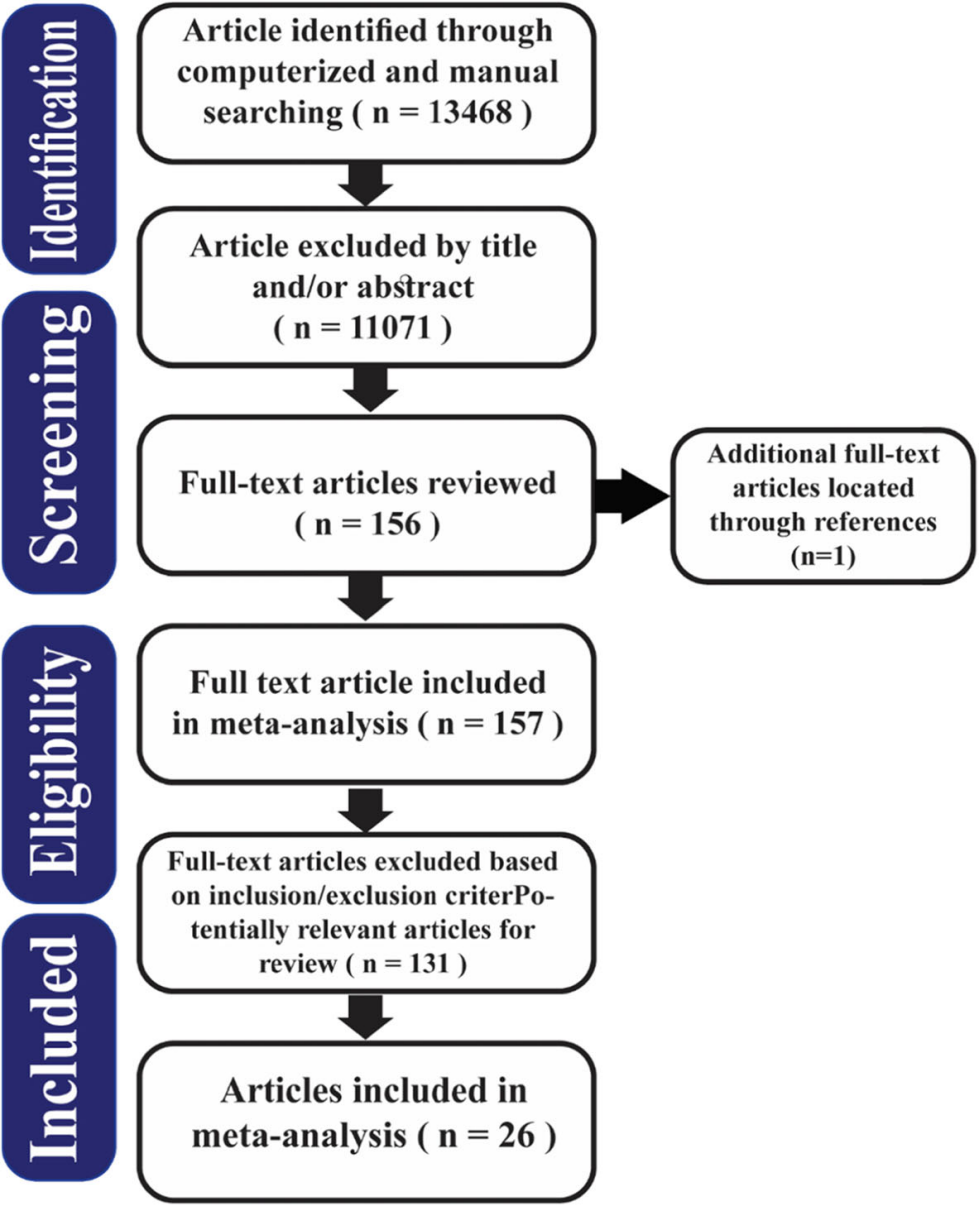
Flow diagram of included /excluded studies

Study characteristics, the risk of bias, and study selection for the included research studies. The selection procedure for the meta-analysis is shown in Fig. 1.

Totally, 13,468 papers were identified. Having excluded 2397 duplicated studies, 11,071 papers were obtained, out of which 10,918 were excluded by title and/or abstract. One hundred fifty-seven full-text papers were opted out and then thoroughly assessed. Finally, 131 unrelated studies were left out and 26 papers were identified eligible for the study. The total number of participants with hospitalization for myocardial infarction was 2,250,473. The largest number of participants was 452,343, which belonged to the study by Milojevic et al., (2014), and the smallest number of participants was 208, which was reported by Akbarzadeh et al., (2018). Considering Berglind et al. (2010), the research was conducted in three cities (Boston, Seattle, and Augsburg), the lag averaging time was 2 h, and the adjusted odds ratio for the PM_2.5_ pollutant was applied in the analysis [39].

**Fig. 2:**
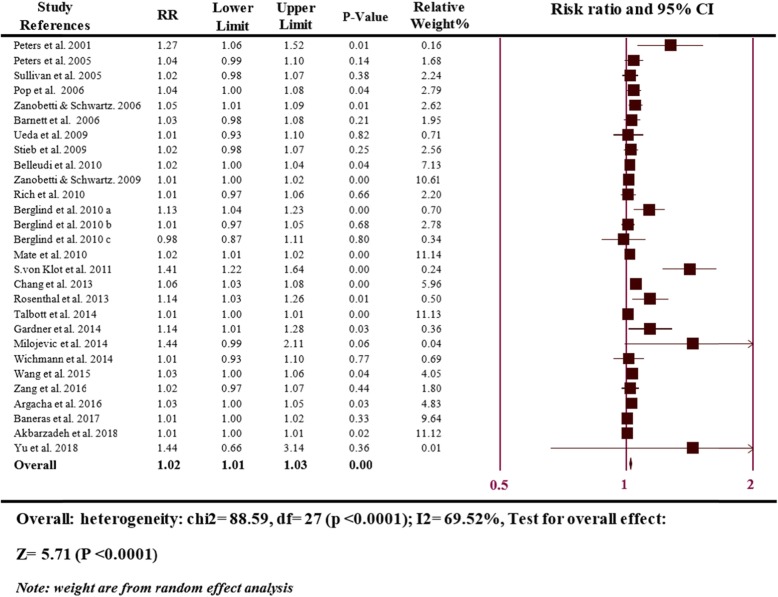
Overall analyses of the effect on the risk of MI hospitalizations associated with a 10 μg/m3 increase in PM_2.5_

Figure 2 is based on 28 comparisons illustrating the association between a 10 μg/m3 increase in the risk of MI and PM_2.5_. The heterogeneity of the research studies was evaluated through random-effects with *P* < 0.0001, and I2 was 69.52%, showing a moderate heterogeneity. The meta-analysis showed a significant positive association between per 10 μg/m3 elevation in PM_2.5_ and MI risk (RR = 1.02; 95% CI 1.01–1.03; *P* ≤ 0.0001) at lag exposure of 0 and 0–1 days.

**Fig. 3:**
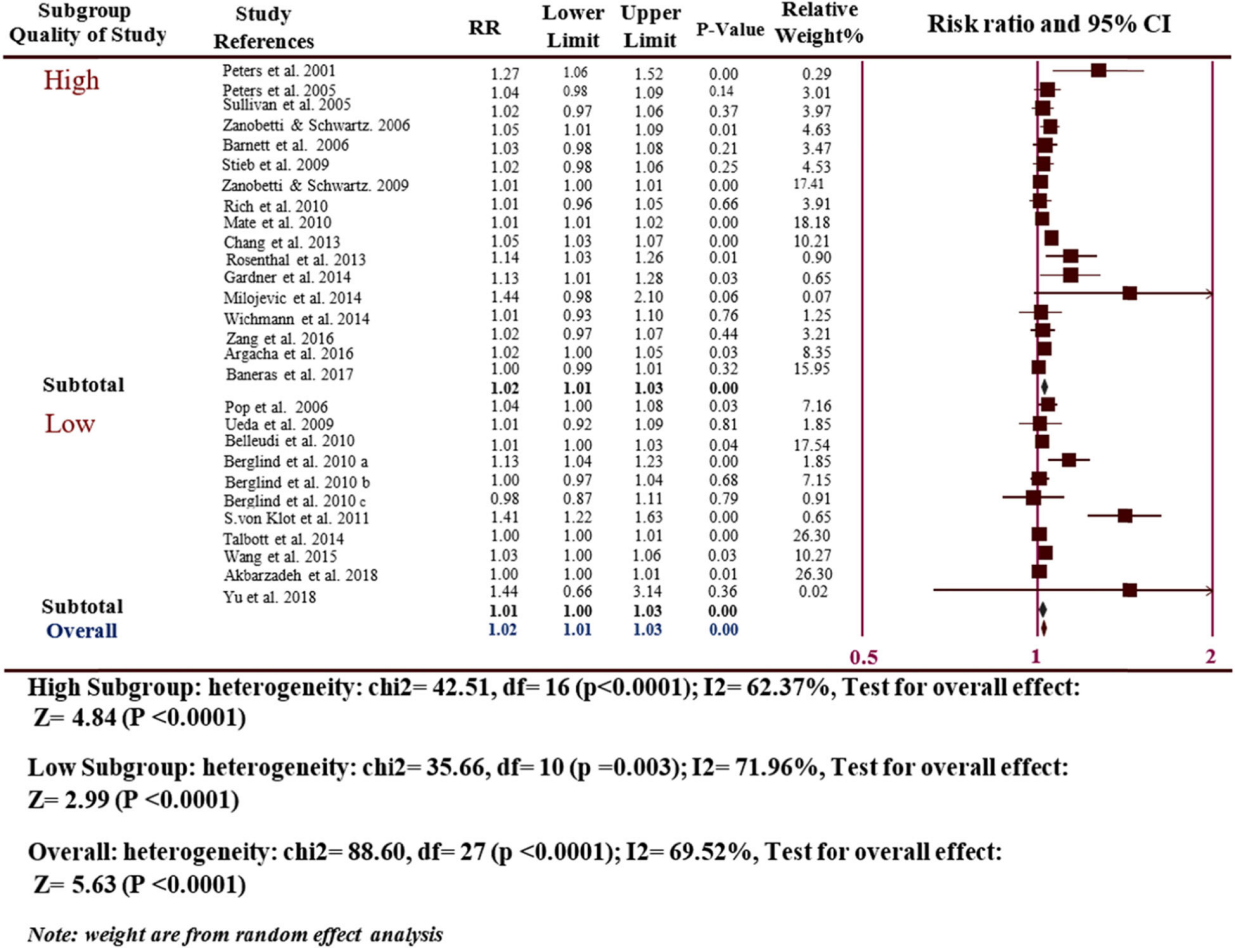
Subgroup analyses of the risk of MI hospitalizations and PM_2.5_ for the quality of study

Considering the quality of study subgroup (17 high-quality studies and 11 low-quality studies), a significantly higher rate of MI risk was seen in high quality studies (RR = 1.02, 95% CI: 1.01–1.03, *P* ≤ 0.0001) with a moderate degree of heterogeneity (I^2^ = 62.37, *P* ≤ 0.0001) and in low quality studies (RR = 1.02, 95% CI: 1.01–1.03, *p* = 0.002) with a moderate to high degree of heterogeneity (I^2^ = 71.96, *P* ≤ 0.0001), which was consistent with the results of the overall analyses (Fig. 3).

**Fig. 4:**
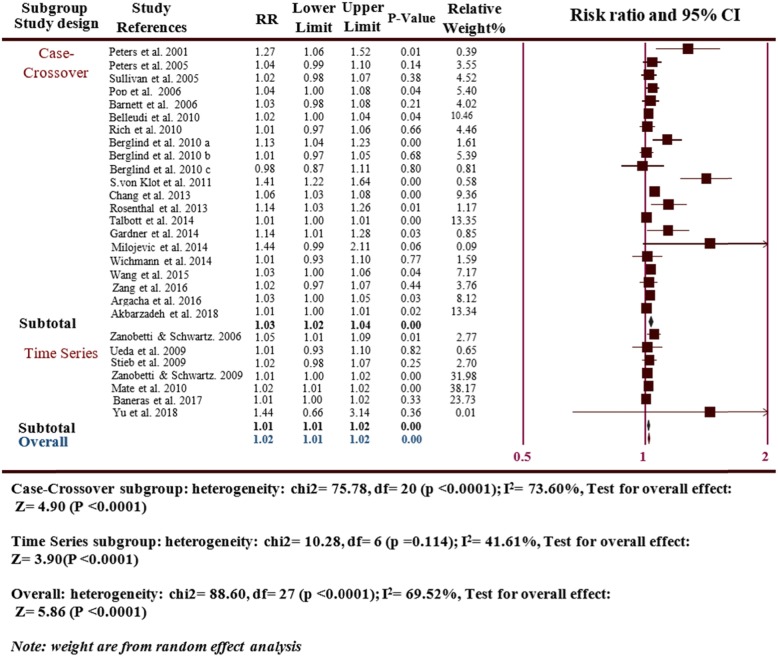
Subgroup analyses the risk of MI hospitalizations and PM_2.5_ for the design

With respect to the study design subgroup, there were a positive association among MI risks in 21 case-crossover studies (RR = 1.03, 95% CI: 1.02–1.04, *p* ≤ 0.0001; I^2^ = 75.78, *P* ≤ 0.0001), which was basically consistent with the overall analyses. There was also statistical significance for 7 time series study subgroup (RR = 1.01, 95% CI: 1.01–1.02, *P* ≤ 0.0001; I^2^ = 41.61, *P* ≤ 0.0001) (Fig. 4).

**Fig. 5:**
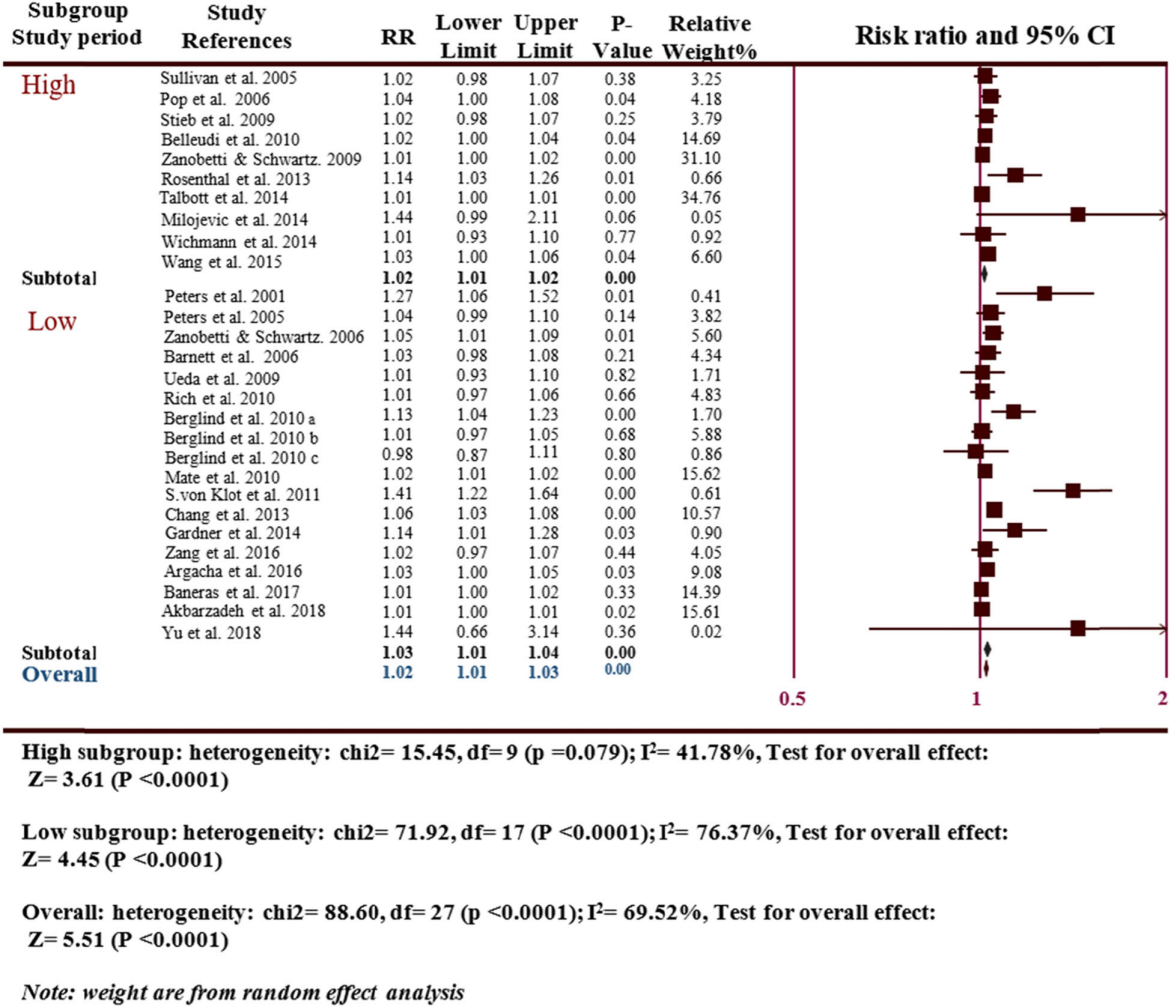
Subgroup analyses the risk of MI hospitalizations and PM_2.5_ for the study period

The subgroup analysis of the study period, Accordingly, the original studies were divided into two subgroups based on the follow-up times: the follow-up of less than 4 years as short study period and the follow-up time of more than 4 years as long study period (10 long study period and 19 short study period) revealed a significantly increased MI risk in the long study period (RR = 1.02, 95% CI: 1.01–1.02, *P* = 0.014) with a moderate degree of heterogeneity (I^2^ = 41.78 *P* ≤ 0.0001) and the short study period (RR = 1.03, 95% CI: 1.01–1.04, *P* ≤ 0.0001) with a moderate degree of heterogeneity (I^2^ = 76.37, *P* ≤ 0.0001), which was consistent with the overall analyses (Fig. 5).

## Discussion

This study aimed to assess the association between exposure to PM_2.5_ and MI hospitalization. The subgroup analyses and the overall analysis were performed based on pooled estimates and relationship between 10 μg/m3 increase in the short-term exposure to PM_2.5_ and the risk of incident MI was pinpointed. Notably, two previous reviews had also shown this association, and in both of them the heterogeneity was supposedly moderate. In an attempt to identify the sources of heterogeneity, Mustafic et al. (2012), formed two subgroups based on study quality and lag exposure, and Luo et al. (2015), formed four subgroups based on study design, study quality, lag exposure, and geographic locations subgroup analyses. However, neither of them could successfully describe the sources of heterogeneity. In the same vein, three subgroup analyses (study quality, study design, and study period) were performed in the present study. According to the results, there was a relatively little difference among the high quality subgroup of the study, the case-crossover study design, and the short study period, all of which contributed to the overall analyses in terms of statistical significance and evidence of heterogeneity. Except for the low quality subgroup of the study which was not statistically significance, the rest were statistically significant. It is assumed that subgroup time series study design and long study period could substantially decrease heterogeneity (I^2^ = 41.61, 41.78). Time-series analysis examined both pre-adjustment and co-adjustment. The pre-adjustment method picks up temporal trends from both the health and air pollution, whereas the co-adjustment approach considers air pollution forecasters and temporal trends [54]. It is presumed that the case-crossover design can create bias as a unidirectional control sampling devoid of time trends [55]. Time-series method is more likely to result in more accurate estimates of risk than the case-crossover method [54]. It is reckoned that the reasons behind the observed heterogeneity in the present study could be the varied design of the included original studies, and also the use of case-crossover and time series studies. Future studies are, nonetheless, expected to use time-series studies, which may help clarify the source of heterogeneity. The follow-up accuracy is also a prerequisite for estimating valid consequences and should be acclaimed systematically. The follow-up index is easy to achieve and could be applied as a reporting criterion for indicators [56]. It is thought that the priority put on the long-term follow-up could enhance the capability to prepare more precise estimates [57]. We also found derivation errors in the second meta-analysis which was performed in 2015. The errors were found in three papers authored by Linn et al. (2000) [58], Xie et al. (2014) [59], and Wichmann et al. (2014), [46]. All these studies might be unintentionally entered the forest plot (PM_2.5_) and did not measure the effect of PM_2.5_ on the risk of incident MI, which could affect the pooled estimates of the study. All these studies might be unintentionally entered the forest plot (PM_2.5_) and did not measure the effect of PM_2.5_ on the risk of Incident MI, which could affect the pooled estimates of the study. In addition, the number of studies identified in the work of Chowdhary et al. (2018), was higher than that of the two previous studies conducted by Mustafic et al. (2012) (13) and Luo et al. (2015) (16). Also, the two reviewers in this study conducted the data extraction phase independently, appraised the papers, investigated all the data, and removed the difference through a third person. Moreover, this study was extensive enough to lower the possibility of publication bias. Even the gray studies were enveloped without any language limitation. This study had some limitations. First, the included original research papers had a great variety and substantially differed from one another in case population, number of people from below 1000 to more than 400,000 people, the city examined, and the study period from under 6 months to over 300 months. Secondly, the assessment of the effect of air pollution on MI could not be well-established as MI is a multifactorial disease associated with diabetes, hypertension, smoking, alcohol, and obesity [60]. Thirdly, the population is chosen in different age group, while the cause of MI in young adults differs from the elderly people. Most of the people with MI are elderly ones with heart problems beforehand. It is estimated that airborne contamination could trigger the undesirable effects to be over-represented for this age group [33, 61].

Finally, as the people who are exposed to a mix combination of air pollutants for longest periods are those at elevated risk of adverse health, outcomes related to individual exposure to a single pollutant cannot be obtained with a high degree of certainty [61].

## Conclusions

The results of this Meta-analysis demonstrated the severity of the relationship between PM_2.5_ and MI with more accurate estimates than analysis presented by 26 studies alone, substantiating the notion that PM_2.5_ levels are key factors in the development of MI hospitalizations. It is highly imperative to conduct further investigations to determine all possible causal relationships and explore potential mechanisms affecting MI. The economic burden of air pollution health-related outcomes is very significant, especially for healthcare providers. Fiscal implications attributed to air pollution are calculated as 253 million to 2.9 billion USD in Asia. Policy makers adopt more effective strategies to help improve air pollution, especially in developing countries, or prevent exposure to PM_2.5_ so as to protect public health.

## Data Availability

All data generated or analyzed during this study are included from preliminary studies are available from the corresponding author on reasonable request.

## Abbreviations

CI: Confidence intervals
Cm2: Comprehensive meta-analysis software version 2
CO: Carbon monoxide
cTn: Cardiac troponin
DALYs: Disability-adjusted Life-years Myocardial infarction
MeSH: Medical Subject Headings
MI: Myocardial infarction
NAAQS: National Ambient Air Quality Standard
NO_2_: Nitrogen dioxide
O_3_: Ozone
OR: Odds ratio
PB: Lead
PM: Particulate matter
PM_10_: Particulate matter < 10 μm aerodynamic diameter
PM_2.5_: Particulate matter < 2.5 μm aerodynamic diameter
PRISMA-P: Preferred reporting items for Systematic review and meta-analysis protocols; RR Relative risk
SO_2_: Sulfur dioxide
SPSS: Statistical Packages for the Social Science
WHO: World Health Organization
YLL: Years of healthy life loss
